# Antioxidant Activity and Phenolic Compounds in Medicinal Plants: A Comparison of Organic and Conventional *Mentha piperita*, *Melissa officinalis*, *Salvia officinalis*, and *Urtica dioica*

**DOI:** 10.3390/molecules30244812

**Published:** 2025-12-17

**Authors:** Dorota Mańkowska, Katarzyna Dems-Rudnicka

**Affiliations:** 1 Institute of Natural Products and Cosmetics, Faculty of Biotechnology and Food Sciences, Lodz University of Technology, Stefanowskiego 2/22, 90-537 Lodz, Poland; 2 Centre of Mathematics and Physics, Lodz University of Technology, Żeromskiego 116, 90-924 Lodz, Poland; katarzyna.dems@p.lodz.pl

**Keywords:** medicinal plants, organic cultivation, conventional cultivation, phenolic compounds, flavonoids, antioxidant activity, phytochemical profile

## Abstract

The study was conducted to determine whether the origin of medicinal plants (conventional vs. organic cultivation) may affect the content of selected bioactive ingredients. This work complements the current state of knowledge on this subject by analysing the content of selected groups of bioactive compounds in four popular herb species (*Mentha piperita*, *Melissa officinalis*, *Salvia officinalis*, *Urtica dioica*). The aim of the study was to compare the total polyphenol (TPC) and flavonoid (TFC) content, antioxidant activity (AA) and phenolic compound profile in herbal extracts of organic and conventional origin. For all species examined, it was demonstrated that water-ethanol extracts from organically grown herbs contain statistically (*p* << 0.05) significantly more TPC, TFC and AA than water-ethanol extracts from conventionally grown herbs. Among the analysed extracts, the highest TPC was found in organic *M. officinalis* (7023.3 mg GAE/100 g d.m.), while the extract of this species from conventional cultivation contained only 3679.4 mg GAE/100 g d.m. TFC in the extracts of organic and conventional *M. piperita* was 1607.6 and 499.4 mg QE/100 g d.m., respectively. Based on GC-MS analysis, between 15 and 25 phenolic compounds were identified, depending on the species of herbal plant. Almost all the identified compounds were phenolic acids. The studies conducted indicate a statistically significantly higher content of antioxidant compounds in herbs from organic farming compared to conventionally grown herbs, and thus their greater health-promoting potential.

## 1. Introduction

Polyphenols are a large and diverse group of plant secondary metabolites that perform a variety of functions, including giving color to flowers, leaves, fruits and seeds, defending plants from microbial and herbivorous attacks, and even protecting delicate plant tissues from strong UV radiation [1,2]. Large amounts of polyphenols can be found in products such as green tea, coffee, cocoa, grapes, chokeberry, blackcurrant fruit, elderberry flowers and fruit, strawberries, raspberries, apples, olives, onion, spinach, ginger, turmeric, rosemary, parsley and many others [3,4]. Plant foods rich in polyphenols are a valuable source of antioxidants in the human diet and reduce the risk of developing many lifestyle diseases [4,5,6,7]. The largest and most thoroughly researched group of polyphenols is flavonoids. Flavonoids have several health-promoting properties, such as alleviating oxidative stress in cells, reducing inflammation by inhibiting the release of pro-inflammatory mediators, and preventing vascular, metabolic, cancerous and neurodegenerative diseases [8]. In addition, some flavonoids have anti-allergic effects by inhibiting the secretion of inflammatory mediators such as histamine (e.g., quercetin, luteolin) [9].

The conventional plant cultivation system, which is the basis of global agricultural production, focuses primarily on maximizing yields and for this purpose uses a variety of chemical plant protection products and mineral soil fertilization. Many of these substances penetrate plant tissues and can potentially harm the health of consumers. Mineral fertilisation, especially with commonly used nitrogen compounds, effectively increases crop yields, but the quality of the crops is not high. Plants grown in soil enriched with mineral fertilisers such as nitrogen produce greater amounts of lush foliage, peptides, proteins, nucleic acids and even some alkaloids, but usually have lower levels of phenolic compounds [10,11,12]. An alternative type of crop cultivation is the organic system, in which the use of mineral fertilisers and chemical plant protection products is prohibited. However, the use of natural fertilisers such as compost or manure, as well as plant extracts and pest control preparations, is permitted. Organic fertilisers have a beneficial effect not only on crops, but also on soil microorganisms [13,14].

Many studies indicate that plant products, mainly vegetables and fruit, from organic farming are of higher quality than those from conventional farming [15,16,17,18,19]. In all described cases, a significantly higher content of phenolic compounds was observed in organic products compared to conventional ones. However, there is still little scientific research in this area concerning medicinal plants. An example is the study by Hallmann and Sabała [20], which compared the content of polyphenols, flavonoids, and phenolic acids in basil, oregano, marjoram, and wild garlic from organic and conventional cultivation. Organically grown herbs were found to contain significantly higher concentrations of all the compounds tested than conventionally grown plants. The higher content of secondary metabolites in plants grown organically is primarily due to the need to activate the plants’ own defence mechanisms against threats from herbivores, soil pathogens and strong ultraviolet radiation. As a result, metabolic pathways for the increased synthesis of defence compounds such as polyphenols, flavonoids, alkaloids and tannins are stimulated [21,22].

Peppermint (*Mentha piperita*) from the *Lamiaceae* family is one of the most popular species of herbal plants, commonly used in the food, cosmetics and pharmaceutical industries. Peppermint herb contains essential oil, significant amounts of phenolic compounds, including rosmarinic, caffeic, ursolic and chlorogenic acids, as well as hesperidin, quercetin, rutin and apigenin, which are responsible for its antioxidant properties. Peppermint has long been used in folk medicine, mainly to treat digestive problems [23]. Lemon balm (*Melissa officinalis*) is a popular species of the *Lamiaceae* family, known for its calming and sleep-inducing properties and its ability to alleviate digestive disorders. The phytochemical composition of lemon balm contains significant amounts of phenolic acids (rosmarinic, caffeic, chlorogenic, ferulic) and flavonoids (luteolin, apigenin) [24]. The same botanical family also includes sage (*Salvia officinalis*), known for its antibacterial and anti-inflammatory properties. In herbal medicine, it is used, among other things, as a remedy for inflammation of the mouth and throat, as well as for digestive problems and excessive sweating. Some of the main secondary metabolites of sage are rosmarinic, ursolic and caffeic acids, as well as flavonoids (luteolin, apigenin, hesperidin) in the form of glycosides [25]. Common nettle (*Urtica dioica*) is a popular herbal plant from the *Urticaceae* family. Nettle is mainly known for its diuretic properties, which support the urinary system. This plant is a rich source of minerals, including iron, which is why it is often used in folk medicine to treat anaemia. In addition to minerals, common nettle is a source of vitamins (A, C, K), phenolic acids (caffeic, *p*-coumaric, protocatechuic, *p*-hydroxybenzoic, ferulic, vanillic), flavonoids (quercetin, luteolin, apigenin, kaempferol) and their derivatives [26].

In recent years, there has been a significant increase in interest in medicinal plants among both consumers and scientists. Herbal plants are a rich source of bioactive compounds, including polyphenols. Many works focus on the properties and phytochemical composition of herbs, but there is still a lack of comparative data between herbs from different crops. The article presents the results of research conducted on four popular species of medicinal plants (*Mentha piperita*, *Melissa officinalis*, *Salvia officinalis*, *Urtica dioica*) of conventional and organic origin. The total polyphenol and flavonoid content, antioxidant activity, and phenolic compound profile of the herbs were determined and discussed in relation to the plant origin.

## 2. Results

### 2.1. Total Polyphenol and Flavonoid Content

To determine the total polyphenol content (TPC) in ethanol extracts of the tested herbs, they were measured spectrophotometrically using the Folin–Ciocalteu reagent. The results obtained, expressed as Gallic Acid Equivalent (GAE) per gram of sample dry mass (mg GAE/100 g d.m.), are presented in Table 1. Furthermore, the total content of the most important subgroup of polyphenols—flavonoids (TFC)—in the tested extracts was determined spectrophotometrically using aluminum chloride (AlCl_3_) (details in the Section 4). The results expressed as Quercetin Equivalent (QE) per gram of sample dry mass (mg QE/100 g d.m.), are presented in Table 1.

The highest TPC was found in organically grown *M. officinalis* (7023.3 mg GAE/100 g d.m.), while in the extract of the same species grown conventionally only 3679.4 mg GAE/100 g d.m. was recorded. A high polyphenol content was also found in organic *M. piperita* extract (3939.8 mg GAE/100 g d.m.). In addition, organically grown peppermint has the highest TFC (1607.6 mg QE/100 g d.m). In turn, the lowest TPC and TFC were found in the extract from conventional *S. officinalis*, 1864.3 mg GAE/100 g d.m. and 274.0 mg QE/100 g d.m., respectively.

### 2.2. Antioxidant Activity

To determine the antioxidant activity of medicinal plant extracts, a test using the DPPH (2,2-diphenyl-1-picrylhydrazyl) radical was performed. The antioxidant activity of the tested plant extracts, expressed as Trolox (6-hydroxy-2,5,7,8-tetramethyl chromane-2-carboxylic acid) Equivalent (TE) per gram of sample dry mass (mg TE/100 g d.m.), is presented in Table 2. The results were also converted into the Radical Scavenging Capacity (RSC) value and are presented in Table 3.

Based on this part of the study, it was observed that among the analyzed herb species, the highest antioxidant activity, expressed as TE and RSC value, was recorded for extracts from *M. officinalis* (2711.6 mg TE/100 g d.m.; RSC = 87.36%) and *M. piperita* (1802.3 mg TE/100 g d.m.; RSC = 59.67%), both of organic origin. In contrast, the lowest antioxidant activity was observed in the extract from *U. dioica* (999.2 mg TE/100 g d.m.; RSC = 28.34%) and *S. officinalis* (1034.8 mg TE/100 g d.m.; RSC = 24.54%), both of conventional origin.

### 2.3. Phenolic Compound Profile

Based on GC-MS analysis, between 15 and 25 phenolic compounds were identified, depending on the species of herbal plant (Table 4). Almost all the identified compounds were phenolic acids. The phenolic acids identified in the highest concentrations are: 3,4-dihydroxyphenyllactic acid (2518.5 mg/100 g d.m. in organic *M. officinalis*), caffeic acid (2423.0 mg/100 g d.m. in organic *M. officinalis*), salicylic acid (491.8 mg/100 g d.m. in organic *U. dioica*), 4-hydroxyphenyllactic acid (373.5 mg/100 g d.m. in organic *S. officinalis*) 3,4-dihydroxyphenylacetic acid (320.6 mg/100 g d.m. in organic *M. piperita*), protocatechuic acid (262.8 mg/100 g d.m. in organic *U. dioica*), ferulic acid (199.8 mg/100 g d.m. in organic *S. officinalis*) and gentisic acid (181.9 mg/100 g d.m. in organic *M. officinalis*).

For all tested herb species, a significantly higher content of identified phenolic compounds was found in organic samples than in conventional ones. For example, the content of caffeic acid was 21.6% higher in the sample of organic *M. piperita* and 50% higher in the sample of organic *S. officinalis* than in their conventional counterparts. The concentration of 3,4-dihydroxyphenyllactic acid, the main phenolic acid in *M. officinalis*, was 43% higher in the organic sample than in the conventional sample. Similarly, samples of *M. piperita* and *M. officinalis* (both organic) were found to have high levels of 3,4-dihydroxyphenylacetic acid, 10.8% and 53% higher than their conventional counterparts, respectively.

In addition to phenolic acids, some samples also contained other compounds, such as vanillin (*S. officinalis*, *U. dioica*, *M. officinalis*), homovanillyl alcohol (*U. dioica*, *M. piperita*), 6,7-dihydroxycoumarin (*U. dioica, M. officinalis*), syringaldehyde (*U. dioica*), tyrosol and eugenol (*M. piperita*). These compounds were found in higher concentrations in organic herbal extracts than in conventional ones, or they were found only in plants of organic origin (tyrosol in *M. piperita*).

## 3. Discussion

Herbs owe their health-promoting properties to the secondary metabolites they contain, including phenolic compounds [4]. In turn, the level of synthesis of these compounds in a plant is the result of its adaptation to environmental conditions, and various biotic and abiotic factors [21,22].

The results presented in our study confirm earlier findings regarding higher levels of secondary metabolites in organically grown medicinal plants than in conventionally grown herbs [20,27]. Our study proves that for all herb species examined, the content of secondary metabolites was significantly higher (*p* << 0.05) in samples from organic farming than in samples from conventional farming.

Among the analyzed species, the highest TPC was obtained for organically grown lemon balm (7023.3 mg/100 g d.m.), followed by peppermint (3939.8 mg/100 g d.m.), also of organic origin. Similar results were obtained in the study by Sadowska et al. [28], in which the antioxidant activity and polyphenol content were examined, among others, in peppermint and lemon balm extracts. According to the authors of the study, no mineral fertilizers or chemical plant protection products were used in the cultivation of herbs. The conditions were therefore like those of an organic plant cultivation system. TPC in the methanol extract of lemon balm was 6648 mg/100 g d.m, while in the methanol extract of peppermint it was 5282 mg/100 g d.m., although it should be noted that the researchers used chlorogenic acid as the standard, while in our study it was gallic acid.

Based on obtained antioxidant activity, the tested herbal samples were arranged in the following order: *M. officinalis* > *M. piperita* > *S. officinalis* > *U. dioica*. Therefore, our results are consistent with those obtained by other researchers who studied antioxidants in 14 species of medicinal plants [29]. On the other hand, authors often used different research methods to determine the antioxidant activity of herbs (ABTS and FRAP), which made it difficult to compare the numerical values with our results. Nevertheless, both in our results and in other studies, lemon balm extract showed the highest antioxidant activity, followed by peppermint extract [28]. Similar results were obtained in a study by Skendi et al. [30], which determined antioxidant activity and phenolic compounds in several species of medicinal plants from the *Lamiaceae* family. Of the five plant species selected for study, *M. officinalis* demonstrated the highest antioxidant activity. The antioxidant activity of plant extracts is closely related to their phenolic compound content. Hence, there is a close correlation between the polyphenol content of the herbs studied and their antioxidant properties, which is clear in the results obtained in our study.

GC-MS analysis identified between 15 and 25 phenolic compounds, depending on the species. The general conclusion from this part of the study corresponds with previous findings, indicating that organically grown herbs contain higher concentrations of bioactive compounds than conventionally grown herbs. Phenolic acids, like flavonoids, are compounds whose structure is based on carbon, and their synthesis in plant cells increases when the soil lacks readily available forms of nitrogen, which is characteristic of organic farming. Therefore, our results support the C/N theory of the activation of different pathways of plant metabolite synthesis depending on carbon and nitrogen availability [31]. Unfortunately, the availability of reference literature sources in which the GC-MS method was used to analyze extracts of selected herb species is very limited, which makes it difficult to compare results.

Among the phenolic compounds identified in the herb samples were 3,4-dihydroxyphenyllactic acid, 3,4-dihydroxyacetic acid, caffeic acid, gentisic acid, salicylic acid, protocatechuic acid and others. The high content of caffeic acid and 3,4-dihydroxyphenylacetic acid, which are hydrolysis products of rosmarinic acid, may indicate its high content, especially in extracts of organic *M. officinalis*, *M. piperita* and *U. dioica*. The results obtained are consistent with those published by other researchers, even though they used different types of chromatographic methods, different extractants and/or different ways of presenting the results [23,25,28,32,33]. For example, in the study by Silva et al. [25], the phytochemical composition of *M. officinalis* and *S. officinalis* was examined using the UPLC method. Twenty-four and fourteen phenolic compounds were identified in *S. officinalis* and *M. officinalis*, respectively. In both cases, the main phenolic acid was rosmarinic acid, in amounts of 22.72 and 40.36 mg/g of extract, respectively.

Protocatechuic acid also had a high share of all tested plant extracts, and, like other identified compounds, it was present in much higher amounts in organic herbal extracts than in conventional herbal extracts, especially in the case of *S. officinalis* and *U. dioica*. The differences observed in the content of individual phenolic compounds in herbs originating from different types of cultivation indicate the key role of factors such as soil conditions, fertilization and biotic stress factors on the intensity of secondary metabolite synthesis in plants.

## 4. Materials and Methods

### 4.1. Plant Material

Four popular species of herbal plants were selected for the study. Medicinal plants in the form of dried leaves were purchased from herbal and food shops in Łódź (Poland). Peppermint (*Mentha piperita*), lemon balm (*Melissa officinalis*), sage (*Salvia officinalis*), and common nettle (*Urtica dioica*) came from organic and conventional farms belonging to two Polish herbal companies. The first one offers products certified from organic farming (PL-EKO-01-001493). The second company offers products from conventional harvests.

### 4.2. Determination of Polyphenol Content

The dried plant material was ground in an electric mill and weighed out to 0.1 g in plastic tubes with stoppers. 5 mL of 80% (*v*/*v*) ethanol (POCH, Gliwice, Poland) was added to each sample. The extraction was carried out for 3 h at 22 °C in a laboratory shaker. Then the extracts were centrifuged (centrifuge MPW Med. Instruments, Warsaw, Poland, MPW-251, 14.000 rpm, 10 min), and the supernatants (extracts) were collected for further studies.

Polyphenol content in the tested herbs was determined using the modified Singleton method [34] using the Folin–Ciocalteu reagent (Sigma-Aldrich, Poznań, Poland). To prepare the reaction mixtures of the tested plant extracts, 50 µL of the extract was mixed with 3.85 mL of distilled water and 100 µL of Folin–Ciocalteu reagent. After mixing the solutions and incubating the samples for 3 min, 1 mL of 10% aqueous disodium carbonate (Na_2_CO_3_; POCH, Gliwice, Poland) solution was added to each sample mixture to obtain an alkaline reaction environment. The samples were mixed thoroughly and incubated for 60 min in the dark place at 22 °C. In parallel, a series of solutions for the standard curve was prepared from gallic acid (POCH, Gliwice, Poland), which was dissolved in 80% (*v*/*v*) ethanol, then diluted from 0.1 to 1.0 mg mL^−1^. The samples were then prepared in a similar manner to the herbal solutions, but instead of the plant extract, 50 µL of the appropriate dilution of gallic acid was used. Then, the absorbance of all samples was measured three times at a wavelength of 760 nm using a double-beam Nicolet Evolution 300 spectrophotometer (Thermo Electron Corporation, Waltham, MA, USA). Total polyphenol content was expressed as mg of Gallic Acid Equivalent per gram of sample dry mass (mg GAE/100 g d.m.).

### 4.3. Determination of Flavonoid Content

Total flavonoid content in the tested herbs was determined using a modified method of Stanojević et al. [35].

To determine the flavonoid content in plant extracts, 500 µL of the extract was mixed with 100 µL of 10% aluminium chloride (AlCl_3_; Chempur, Piekary Śląskie, Poland) solution, 100 µL of 1 M sodium acetate (CH_3_COONa; Chempur, Piekary Śląskie, Poland) solution and 4.3 mL of distilled water. The mixture was then incubated in the dark place for 40 min at 22 °C. In parallel, a series of solutions for the standard curve was prepared from quercetin (Sigma-Aldrich, Poznań, Poland), which was dissolved in 80% (*v*/*v*) ethanol, with dilutions ranging from 0.01 to 0.1 mg/mL. The samples for the standard curve were then prepared in a similar manner to the herbal solutions, but instead of the plant extract, 500 µL of the appropriate quercetin dilution was used. Then, the absorbance of all samples was measured three times at a wavelength of 425 nm using a double-beam Nicolet Evolution 300 spectrophotometer. The total flavonoid content was expressed based on the calibration curve as mg of Quercetin Equivalent per gram of sample dry mass (mg QE/100 g d.m.).

### 4.4. Determination of Antioxidant Activity by the DPPH Radical Reduction Method

The antioxidant activity of plant extracts was determined according to the modified method of Brand-Williams et al. [36] using the DPPH radical. A 0.11 mM DPPH (Merck Life Science, Poznań, Poland) solution was prepared in 96% (*v*/*v*) ethanol. The test sample was a mixture of 3 mL of DPPH solution and 20 µL of ethanolic plant extract. The control sample was prepared by mixing 3 mL of DPPH solution and 20 µL of ethanol. The samples were then incubated for 30 min in the dark at 22 °C, and their absorbance was measured three times at a wavelength of 517 nm against pure ethanol (blank) using a double-beam UV-VIS spectrophotometer Nicolet Evolution 300. Samples for the standard curve were prepared for a solution of Trolox (Merck Life Science, Poznań, Poland) in 96% (*v*/*v*) ethanol, with concentrations ranging from 0.1 to 2.0 mM. The method of preparing solutions for the standard curve and performing the analysis was analogous to that for the plant samples. The antioxidant activity of the tested extracts was calculated based on the equation of the calibration curve and expressed as Trolox Equivalent (mg TE/100 g d.m.). The results were also converted into the radical scavenging capacity (RSC) value, i.e., the percentage effectiveness of DPPH radical removal by plant extracts, calculated according to the following equation:

RSC [%] = [(A_0_ − A)/A_0_] × 100

RSC—radical scavenging capacity; A_0_—absorbance of the control sample; A—absorbance of the plant sample.

### 4.5. Phenolic Compound Profile

To identify phenolic compounds in the tested plant samples, a modified method developed by Cyran et al. [37] was used. In the first step, 2 mL of the herbal extract was mixed with 20 µL of 6 M HCl (POCH, Gliwice, Poland) to conduct acidic hydrolysis of the glycosidic compounds. The samples were then evaporated to dryness at 45 °C for approximately 10 h. In the next step, 3 mL of ethyl acetate (Sigma-Aldrich, Poznań, Poland) was added to the dry sample and shaken. 0.2 mL of this solution was transferred to a glass chromatography vial and evaporated to dryness under a gentle stream of nitrogen. In the next step, 0.2 mL of bis(trimethylsilyl)trifluoroacetamide with trimethylchlorosilane (BSTFA + TMCS; Merck KGaA, Darmstadt, Germany) (99:1) was added to the vial and heated at 80 °C for 2 h. GC-MS analysis was performed on a LECO Pegasus 4D instrument (LECO Corporation, St. Joseph, MI, USA), equipped with an Agilent 7890A gas chromatograph (Agilent, Santa Clara, CA, USA), coupled with a mass spectrometer with a time-of-flight analyzer.

### 4.6. Statistical Analysis

The results are presented as means ± SD with n=3 replicates. Assumptions regarding normal distributions in individual groups and homogeneity of variance were fulfilled. Differences in the content of the tested compounds for organic and conventional cultivation of the four examined species of herbs were tested using Student’s *t*-test, with groups considered different when *p* < 0.05 was obtained (*p* << 0.05 was obtained in each test). A combined analysis of the content of the studied compounds by cultivation method and herb species was performed using an ANOVA test followed by Tukey’s post hoc tests at a significance level of 0.05. Groups marked with the same letter were not significantly different. Statistical analysis was performed using R software version 4.5.1. (R Core Team,, R Foundation for Statistical Computing, Vienna, Austria, 2025).

## 5. Conclusions

For all medicinal plant species studied, it was statistically demonstrated (*p* << 0.05) that the polyphenol and flavonoid content, as well as the antioxidant activity of herbs from organic cultivation is significantly higher than in herbs from conventional cultivation. The highest antioxidant potential was obtained for *M. officinalis* and secondly for *M. piperita*, both from organic farming, which corresponds to the highest content of polyphenols and flavonoids in the same species. *S. officinalis* from conventional cultivation showed the lowest antioxidant activity. These observations were confirmed by GC-MS analysis, which identified between 15 and 25 phenolic compounds, depending on the species, with significantly higher concentrations in organically grown herbs than in conventionally grown herbal plants. Our research has confirmed that conventional herb cultivation methods result in lower levels of phenolic compounds than in herbs grown using organic methods, which is consistent with the C/N theory.

The medicinal plant species selected for this study are a rich source of antioxidant compounds, especially herbs from organic cultivation. The obtained results are important due to their usefulness in further research on medicinal plants, factors influencing their phytochemical composition, and potential applications in the food, cosmetics, and pharmaceutical industries.

## Figures and Tables

**Table 1:** Total polyphenol (TPC) [mg GAE/100 g d.m.] and flavonoid (TFC) [mg QE/100 g d.m.] content in the tested medicinal plants depending on their origin *.

Group of Compounds	Plant Origin	*Mentha* *piperita*	*p*-Value	*Melissa* *officinalis*	*p*-Value	*Salvia* *officinalis*	*p*-Value	*Urtica* *dioica*	*p*-Value
TPC	organic	3939.8 ^b^ ± 0.1	5.21 × 10^−16^	7023.3 ^a^ ± 0.1	1.77 × 10^−7^	3091.4 ^bc^ ± 3.1	1.05 × 10^−6^	2675.9 ^bc^ ± 0.1	2.55 × 10^−6^
	conventional	3228.7 ^bc^ ± 0.1		3679.4 ^b^ ± 3.5		1864.3 ^c^ ± 0.1		1931.4 ^c^ ± 2.9	
TFC	organic	1607.6 ^A^ ± 0.6	4.54 × 10^−14^	1050.9 ^BC^ ± 1.2	1.04 × 10^−11^	637.6 ^CDE^ ± 0.6	3.42 × 10^−12^	1212.6 ^AB^ ± 0.7	1.11 × 10^−11^
	conventional	499.4 ^DE^ ± 0.3		511.5 ^DE^ ± 0.6		274.0 ^E^ ± 0.3		775.5 ^BCD^ ± 0.8	

* The results are presented as means (±) standard deviation (SD); the number of sample *n* = 3; significant level *α* = 0.05; *p*-value presented regards *t*-tests among two cultivation methods for each species with H_a_: the content of examined group of compounds for herbs from organic origin is higher; mean values with different letters (^a–c^ for TPC or ^A–E^ for TFC) are statistically different in Tukey’s tests.

**Table 2:** Antioxidant activity of the tested medicinal plants depending on their origin expressed as Trolox equivalent [mg TE/100 g d.m.] *.

Plant Origin	*Mentha* *piperita*	*p*-Value	*Melissa* *officinalis*	*p*-Value	*Salvia* *officinalis*	*p*-Value	*Urtica dioica*	*p*-Value
organic	1802.3 ^b^ ± 2.5	5.89 × 10^−9^	2711.6 ^a^ ± 0.1	3.17 × 10^−6^	1609.2 ^bc^ ± 1.3	1.63 × 10^−10^	1285.9 ^cd^ ± 1.2	6.42 × 10^−9^
conventional	1558.1 ^bc^ ± 1.3		1899.8 ^b^ ± 3.6		1034.8 ^d^ ± 2.4		999.2 ^d^ ± 3.2	

* The results are presented as means (±) standard deviation (SD); the number of sample *n* = 3; significant level *α* = 0.05; *p*-value presented regards *t*-tests among two cultivation methods for each species with H_a_: the content of examined compounds for herbs from organic origin is higher; mean values with different letters (^a–d^) are statistically different in Tukey’s tests.

**Table 3:** Antioxidant activity of the tested medicinal plants depending on their origin expressed as percentage of DPPH radical scavenging (RSC) [%].

Plant Origin	*Mentha piperita*	*Melissa* *officinalis*	*Salvia* *officinalis*	*Urtica* *dioica*
organic	59.67	87.36	48.35	41.80
conventional	46.71	58.47	24.54	28.34

**Table 4:** The average content of phenolic compounds [mg/100 g d.m.] identified by GC-MS in the tested medicinal plants depending on their origin *.

Compound	*Mentha piperita*		*Melissa officinalis*		*Salvia officinalis*		*Urtica dioica*	
	Organic	Conventional	Organic	Conventional	Organic	Conventional	Organic	Conventional
2-Hydroxymandelic acid	ND	ND	ND	ND	ND	ND	6.1 ± 0.3	3.4 ± 0.2
2,3-Dihydroxybenzoic acid	ND	ND	11.6 ± 0.7	5.2 ± 0.3	ND	ND	ND	ND
3,4-Dihydroxymandelic acid	ND	ND	17.0 ± 0.9	8.7 ± 0.6	ND	ND	ND	ND
2,3-Dimethoxybenzoic acid	7.0 ± 0.4	6.0 ± 0.3	ND	ND	ND	ND	ND	ND
3,4,5-Trimethoxybenzoic acid	1.2 ± 0.1	1.6 ± 0.1	ND	ND	ND	ND	ND	ND
3,4-Dihydroxyphenylacetic acid	320.6 ± 11.8	286.1 ± 11.4	106.2 ± 4.3	49.9 ± 2.8	13.5 ± 0.8	7.3 ± 0.3	ND	ND
3,4-Dihydroxyphenyllactic acid	1281.9 ± 29.2	1043.3 ± 35.5	2518.5 ± 126.4	1435.6 ± 73.8	584.4 ± 30.1	315.6 ± 15.6	52.1 ± 2.9	32.3 ± 1.7
3,4-Dimethoxybenzoic acid	15.6 ± 0.9	11.1 ± 0.6	ND	ND	ND	ND	ND	ND
3-Hydroxybenzoic acid	ND	ND	ND	ND	ND	ND	8.0 ± 0.5	5.1 ± 0.4
3-Hydroxycinnamic acid	1.5 ± 0.1	ND	ND	ND	ND	ND	ND	ND
4-Hydroxybenzaldehyde	ND	ND	ND	ND	ND	ND	2.3 ± 0.1	1.5 ± 0.1
4-Hydroxybenzoic acid	ND	ND	35.9 ± 2.3	18.0 ± 0.9	60.7 ± 3.1	34.0 ± 1.6	ND	ND
4-Hydroxymandelic acid	1.4 ± 0.0	ND	ND	ND	ND	ND	ND	ND
4-Hydroxyphenylacetic acid	ND	ND	47.4 ± 2.5	21.8 ± 1.2	16.3 ± 0.7	9.1 ± 0.5	30.6 ± 1.3	19.3 ± 0.8
4-Hydroxyphenyllactic acid	ND	ND	31.4 ± 1.9	17.3 ± 1.3	373.5 ± 17.9	231.6 ± 11.1	ND	ND
6,7-Dihydroxycoumarin	ND	ND	5.2 ± 0.6	2.9 ± 0.2	ND	ND	5.8 ± 0.3	3.3 ± 0.2
Caffeic acid	1484.9 ± 35.5	1164.2 ± 45.7	2423.0 ± 117.3	1284.2 ± 66.3	637.3 ± 31.7	318.6 ± 16.1	1429.4 ± 73.1	743.3 ± 35.8
Dihydrocaffeic acid	ND	ND	ND	ND	ND	ND	12.4 ± 0.7	6.4 ± 0.4
Eugenol	1.9 ± 0.1	2.2 ± 0.1	ND	ND	ND	ND	ND	ND
Ferulic acid	46.0 ± 1.5	39.5 ± 1.7	29.2 ± 1.7	15.8 ± 0.9	199.8 ± 8.3	105.9 ± 5.3	71.7 ± 3.4	39.4 ± 1.8
Gallic acid	13.8 ± 0.4	9.8 ± 0.4	5.3 ± 0.5	2.7 ± 0.1	ND	ND	39.5 ± 2.0	21.8 ± 1.2
Gentisic acid	95.3 ± 3.9	81.0 ± 3.5	181.9 ± 9.4	83.7 ± 4.3	31.8 ± 1.2	19.4 ± 0.8	168.3 ± 8.3	95.9 ± 4.9
Homoprotocatechuic acid	22.2 ± 1.0	25.4 ± 1.0	ND	ND	ND	ND	ND	ND
Homovanillyl alcohol	18.6 ± 0.6	17.3 ± 0.9	ND	ND	ND	ND	19.4 ± 0.8	10.5 ± 0.4
Hydrocaffeic acid	14.6 ± 0.7	11.4 ± 0.6	ND	ND	ND	ND	ND	ND
Hydrocinnamic acid	ND	ND	39.9 ± 2.1	23.1 ± 1.4	ND	ND	ND	ND
Isoferulic acid	ND	ND	ND	ND	ND	ND	4.4 ± 0.3	2.8 ± 0.1
*m*-Coumaric acid	3.2 ± 0.1	2.6 ± 0.2	3.5 ± 0.8	1.9 ± 0.2	12.6 ± 0.7	6.7 ± 0.3	ND	ND
*p*-Anisic acid	2.9 ± 0.1	ND	ND	ND	ND	ND	ND	ND
*p*-Coumaric acid	17.0 ± 0.7	12.3 ± 0.6	44.5 ± 2.4	24.9 ± 1.2	44.3 ± 1.9	26.1 ± 1.2	36.3 ± 1.9	20.3 ± 1.0
*p*-Hydroxyhydrocinnamic acid	ND	ND	ND	ND	ND	ND	11.8 ± 0.7	7.0 ± 0.4
*p*-Methoxycinnamic acid	1.4 ± 0.1	1.9 ± 0.1	ND	ND	ND	ND	ND	ND
Protocatechuic acid	220.7 ± 5.3	203.9 ± 8.7	218.9 ± 10.1	120.4 ± 6.2	122.8 ± 4.5	60.2 ± 2.9	262.8 ± 12.1	136.6 ± 6.5
Salicylic acid	54.6 ± 2.3	53.6 ± 2.8	149.2 ± 8.1	85.0 ± 4.6	148.8 ± 7.6	87.8 ± 4.1	491.8 ± 24.7	309.8 ± 16.8
Syringic acid	28.0 ± 1.3	24.3 ± 1.3	13.1 ± 0.8	7.5 ± 0.6	35.5 ± 1.4	18.1 ± 0.7	ND	ND
Syringaldehyde	ND	ND	ND	ND	ND	ND	1.2 ± 0.1	0.7 ± 0.1
Tyrosol	6.1 ± 0.3	ND	ND	ND	ND	ND	ND	ND
Vanillic acid	22.8 ± 1.0	21.3 ± 0.9	18.8 ± 1.1	9.2 ± 0.5	68.5 ± 3.6	39.8 ± 2.1	12.1 ± 0.8	6.4 ± 0.4
Vanillin	ND	ND	1.0 ± 0.3	0.5 ± 0.2	89.1 ± 4.3	43.7 ± 2.0	3.2 ± 0.2	1.7 ± 0.1
β-resorcylic acid	8.6 ± 0.4	7.6 ± 0.4	ND	ND	ND	ND	ND	ND

* The results are presented as means (±) standard deviation (SD); the number of samples *n* = 2; abbreviation: ND—Not Detected.

## Data Availability

The original contributions presented in this study are included in the article. Further inquiries can be directed at the corresponding author.

## References

[1] Quideau S., Deffieux D., Douat-Casassus C., Pouységu L. (2011). Plant polyphenols: Chemical properties, biological activities, and synthesis. Angew. Chem. Int. Ed. Engl..

[2] Lang Y., Gao N., Zang Z., Meng X., Lin Y., Yang S., Yang Y., Jin Z., Li B. (2024). Classification and antioxidant assays of polyphenols: A review. J. Future Foods.

[3] Pérez-Jiménez J., Neveu V., Vos F., Scalbert A. (2010). Identification of the 100 richest dietary sources of polyphenols: An application of the Phenol-Explorer database. Eur. J. Clin. Nutr..

[4] Pandey K.B., Rizvi S.I. (2009). Plant polyphenols as dietary antioxidants in human health and disease. Oxid. Med. Cell Longev..

[5] Iqbal I., Wilairatana P., Saqib F., Nasir B., Wahid M., Latif M.F., Iqbal A., Naz R., Mubarak M.S. (2023). Plant polyphenols and their potential benefits on cardiovascular health: A review. Molecules.

[6] Guo J., Li K., Lin Y., Liu Y. (2023). Protective effects and molecular mechanisms of tea polyphenols on cardiovascular diseases. Front. Nutr..

[7] Quiñones M., Miguel M., Aleixandre A. (2013). Beneficial effects of polyphenols on cardiovascular disease. Pharmacol. Res..

[8] Jomova K., Alomar S.Y., Valko R., Liska J., Nepovimova E., Kuca K., Valko M. (2025). Flavonoids and their role in oxidative stress, inflammation, and human diseases. Chem. Biol. Interact..

[9] Rakha A., Umar N., Rabail R., Butt M.S., Kieliszek M., Hassoun A., Aadil R.M. (2022). Anti-inflammatory and anti-allergic potential of dietary flavonoids: A review. Biomed. Pharmacother..

[10] Albornoz F. (2016). Crop responses to nitrogen overfertilization: A review. Sci. Horticult..

[11] Amtmann A., Armengaud P. (2009). Effects of N, P, K and S on metabolism: New knowledge gained from multi-level analysis. Curr. Opin. Plant Biol..

[12] Bourn D., Prescott J. (2002). A comparison of the nutritional value, sensory qualities, and food safety of organically and conventionally produced foods. Crit. Rev. Food Sci. Nutr..

[13] Luo Y., van Veelen H.P.J., Chen S., Sechi V., ter Heijne A., Veeken A., Buisman C.J.N., Bezemer T.M. (2022). Effects of sterilization and maturity of compost on soil bacterial and fungal communities and wheat growth. Geoderma.

[14] Adugna G. (2016). A review on impact of compost on soil properties, water use and crop productivity. Acad. Res. J. Agri. Sci. Res..

[15] Young J.E., Zhao X., Carey E.E., Welti R., Yang S.-S., Wang W. (2005). Phytochemical phenolics in organically grown vegetables. Mol. Nutr. Food Res..

[16] Raigón M.D., Rodríguez-Burruezo A., Prohens J. (2010). Effects of organic and conventional cultivation methods on composition of eggplant fruits. J. Agric. Food Chem..

[17] Carbonaro M., Mattera M., Stefano N., Bergamo P., Cappelloni M. (2002). Modulation of antioxidant compounds in organic vs conventional fruit (peach, *Prunus perlica* L., and pear, *Prunus communis* L.). J. Agric. Food Chem..

[18] Stracke B.A., Rufer C.E., Weibel F.P., Bubv A., Watzl B. (2009). Three-year comparison of the polyphenol contents and antioxidant capacities in organically and conventionally produced apples (*Malus domestica* Bork. cultivar ‘Golden Delicious’). J. Agric. Food Chem..

[19] Anttonen M.J., Karjalainen R.O. (2006). High-performance liquid chromatography analysis of black currant (*Ribes nigrum* L.) fruit phenolics grown either conventionally or organically. J. Agric. Food Chem..

[20] Hallmann E., Sabała P. (2020). Organic and conventional herbs quality reflected by their antioxidant compounds concentration. Appl. Sci..

[21] Saini N., Anmol A., Kumar S., Wani A.W., Bakshi M., Dhiman Z. (2024). Exploring phenolic compounds as natural stress alleviators in plants—A comprehensive review. Physiol. Mol. Plant Pathol..

[22] Qaderi M.M., Martel A.B., Strugnell C.A. (2023). Environmental factors regulate plant secondary metabolites. Plants.

[23] Cavar Zeljković S., Šišková J., Komzáková K., De Diego N., Kaffková K., Tarkowski P. (2021). Phenolic Compounds and Biological Activity of Selected Mentha Species. Plants.

[24] Miraj S., Kopaei R., Kiani S. (2017). *Melissa officinalis* L: A Review Study with an Antioxidant Prospective. J. Evid. Based Complement. Altern. Med..

[25] Silva B.N., Cadavez V., Caleja C., Pereira E., Calhelha R.C., Añibarro-Ortega M., Finimundy T., Kostić M., Soković M., Teixeira J.A. (2023). Phytochemical Composition and Bioactive Potential of *Melissa officinalis* L., *Salvia officinalis* L. and *Mentha spicata* L. Extracts. Foods.

[26] Durović S., Kojić I., Radić D., Smyatskaya Y.A., Bazarnova J.G., Filip S., Tosti T. (2024). Chemical Constituents of Stinging Nettle (*Urtica dioica* L.): A Comprehensive Review on Phenolic and Polyphenolic Compounds and Their Bioactivity. Int. J. Mol. Sci..

[27] Kazimierczak R., Hallmann E., Rembiałkowska E. (2015). Effects of organic and conventional production systems on the content of bioactive substances in four species of medicinal plants. Biol. Agric. Hortic..

[28] Sadowska U., Armenta Villavicencio R., Dziadek K., Skoczylas J., Sadowski S.K., Kopeć A. (2024). The identification of polyphenolic compounds and the determination of antioxidant activity in extracts and infusions of peppermint, lemon balm and lavender. Appl. Sci..

[29] Ulewicz-Magulska B., Wesolowski M. (2023). Antioxidant Activity of Medicinal Herbs and Spices from Plants of the *Lamiaceae*, *Apiaceae* and *Asteraceae* Families: Chemometric Interpretation of the Data. Antioxidants.

[30] Skendi A., Irakli M., Chatzopoulou P. (2017). Analysis of phenolic compounds in Greek plants of Lamiaceae family. J. App. Res. Med. Aromat. Plants.

[31] Bryant J.P., Chapin F.S., Klein D.R. (1983). Carbon/nutrient balance of boreal plants in relation to vertebrate herbivory. Oikos.

[32] Jasicka-Misiak I., Poliwoda A., Petecka M., Buslovych O., Shlyapnikov V.A., Wieczorek P.P. (2018). Antioxidant phenolic compounds in *Salvia officinalis* L. and *Salvia sclarea* L.. Ecol. Chem. Eng. S..

[33] Sahal A., Hussain A., Kumar S., Dobhal A., Ahmad W., Chand K., Richa R., Lohani U.C. (2025). Nettle (*Urtica dioica*) leaves as a novel food: Nutritional, phytochemical profiles, and bioactivities. Food Chem. X.

[34] Singleton V.L., Orthofer R., Lamuela-Raventos R.M. (1999). Analysis of total phenols and other oxidation substrates and antioxidants by means of folin–ciocalteu reagent. Meth. Enzymol..

[35] Stanojević L., Stanković M., Nikolić V., Nikolić L., Ristić D., Canadanovic-Brunet J., Tumbas V. (2009). Antioxidant activity and total phenolic and flavonoid contents of *Hieracium pilosella* L. extracts. Sensors.

[36] Brand-Williams W., Cuvelier M.E., Berset C. (1995). Use of a free radical method to evaluate antioxidant activity. LWT Food Sci. Technol..

[37] Cyran M.R., Dynkowska W.M., Ceglińska A., Bonikowski R. (2021). Improving rye bread antioxidant capacity by bread-making methodology: Contribution of phosphate-buffered saline- and methanol-soluble phenolic phytochemicals with different molecular profiles. J. Cereal Sci..

